# End-of-life pathology in UM-HET3 mice treated with 16 α‑hydroxyestradiol or late‑start canagliflozin

**DOI:** 10.1007/s11357-025-01741-3

**Published:** 2025-07-02

**Authors:** Jessica M. Snyder, David E. Harrison, Ron Korstanje, Brett Ginsburg, Peter C. Reifsnyder, James Nelson, Scott Leiser, Catherine Kaczorowski, Denise M. Imai, Adam B. Salmon, Randy Strong, Warren Ladiges, Richard A. Miller

**Affiliations:** 1 Department of Comparative Medicine, School of Medicine, University of Washington, Seattle, WA USA; 2 The Jackson Laboratory, Bar Harbor, ME USA; 3 Barshop Institute for Longevity and Aging Studies and Department of Pharmacology, The University of Texas Health Science Center, San Antonio, TX USA; 4 Barshop Institute for Longevity and Aging Studies and Departments of Molecular Medicine and Cellular and Integrative Physiology, The University of Texas Health Science Center, San Antonio, TX USA; 5 Department of Molecular & Integrative Physiology and Geriatrics Center, University of Michigan, Ann Arbor, MI USA; 6 Department of Neurology and Geriatrics Center, University of Michigan, Ann Arbor, MI USA; 7 Department of Pathology, Microbiology and Immunology, University of California, Davis, CA USA; 8 Geriatric Research, Education and Clinical Center and Research Service, South Texas Veterans Health Care System, San Antonio, TX USA; 9 Department of Pathology and Geriatrics Center, University of Michigan, Ann Arbor, MI USA; 10 Barshop Institute for Longevity and Aging Studies and Department of Psychiatry, The University of Texas Health Science Center at San Antonio, San Antonio, TX USA

**Keywords:** Canagliflozin, Pathology, Cause of death

## Abstract

Canagliflozin (Cana) started  at 16 months of age and 16-hydroxy-estradiol (OH_Est) started at 12 months each led to significant increases in lifespan in male UM-HET3 mice but significant decreases in female lifespan. To seek insights into the basis for these sex-specific effects, we performed end-of-life histopathological analyses of control and treated mice for all three interventions testing program sites. There were no significant drug-induced alterations in inferred cause of death, although statistical power was low for such comparisons. Tabulation of incidental lesions (i.e., combining lethal and non-lethal lesions) revealed a complex set of significant and near-significant changes caused by each of the two agents, in some cases absent, or even opposite in direction, in one of the two sexes. The analysis did not, however, reveal a clear pattern that would explain the selective sex-specific effects of either agent on lifespan. It is plausible that the female-specific harm induced by each of these agents could reflect harmful or toxic effects that are not easily detectable by histopathological examination.

**Supplementary Information:**

The online version contains supplementary material available at 10.1007/s11357-025-01741-3.

## Introduction

Genetically heterogeneous UM-HET3 mice have been used to evaluate a variety of compounds for life-extending benefits. Recently, our group, the Interventions Testing Program (ITP), tested two agents, 16α-hydroxyestradiol (OH_Est), started at 12 months of age, and Canagliflozin (Cana), started at 16 months of age (Cana_16). Each drug resulted in a significant increase in median lifespan in male mice of approximately 15%, as we have recently reported [1]. However, each drug also led to a significant decrease in median lifespan in female mice, at 7% for OH_Est and 6% for Cana_16 [1]. In two previous studies, Cana fed from an earlier age (6–7 months) increased male lifespan without significantly changing female lifespan [1, 2]. In other previous studies, 17-α-estradiol fed to mice from an early age at two different doses also preferentially increased male but not female lifespan [3, 4].

There is little information on the effects of long-term administration of OH_Est to mice. Investigation of this drug by the ITP was prompted by the previous results showing an improvement in lifespan and healthspan in male mice treated with 17-α-estradiol [3, 4], and evidence that this drug undergoes metabolism to other estrogens in male mice only. Because 17-α-estradiol undergoes sex-specific metabolism to estriol-3-sulfate and 16-oxoestradiol 3-sulfate, which are products of the conversion of 17aE2 to estriol, in male mice, we hypothesized that treating mice directly with estriol might therefore prove beneficial in both sexes [1]. For this reason, we selected 16α-hydroxyestradiol (OH_Est) as a test agent.

Canagliflozin is an oral hypoglycemic drug and SGLT2 inhibitor for the treatment of diabetes, which has shown to reduce a number of age-associated lesions in male mice [5], without a significant positive or negative effect in female mice. Investigation of other lifespan-extending interventions such as rapamycin [6] has shown a benefit even when started later in life, and we determined that this is true in male mice for Cana as well [1].

In this report, we examined cause of death of male and female mice treated with OH_Est and late onset Cana and assessed the presence and severity of age-associated lesions which might be associated with treatment in either male or female mice.

## Methods

### Mice

Lifespan data for mice included in this study has been previously reported, and details of mouse generation and husbandry are included in that publication [1]. Briefly, these UM-HET3 mice are the progeny of CByB6F1 mothers (JAX stock 100009) and C3D2F1 fathers (JAX stock 100004). Mice were included from three test sites: the Jackson Laboratory (TJL), the University of Michigan (UM), and the University of Texas Health Science Center at San Antonio (UT). Mice were either treated with control diet, with diet including 5 ppm of 16-α-hydroxyestradiol from 12 months of age, or diet including 180 ppm of canagliflozin from 16 months of age. Mice underwent no experimental manipulation other than being weighed at 6, 12, 18, and 24 months of age. Mice were maintained until “end of life”, which included mice found dead in the cage or mice found in a moribund state and therefore euthanized per the IACUC protocol. All the experiments were approved by the respective animal care committees at each of the three sites.

### Pathology

After mice were found dead or euthanized, incisions were made into the abdominal cavity, thoracic cavity, and cranium. Mice were then submerged in 10% neutral buffered formalin. Random cases from each of the three sites were then sent to the University of Washington for gross inspection and for preparation of slides for histological examination in a blinded fashion. Histology was not performed on one mouse due to extensive autolysis. 164 total mice were examined grossly and histologically including 29 female mice treated with OH_EST; 29 male mice treated with OH_EST; 30 female mice treated with Cana_16; 31 male mice treated with Cana_16; 30 control female mice; and 15 control male mice.

Gross necropsy of the fixed mice was performed, and organs were removed and trimmed for histology. Organs examined histologically included: decalcified cross section of skull with brain at the level of the pituitary; lungs; liver; kidneys; heart; pancreas; spleen; mesenteric fat; lymph node; uterus and ovaries or testes, epididymis, and seminal vesicles); adrenal glands; thyroid; and any masses or abnormalities noted grossly. Tissues were routinely processed and paraffin embedded, and 4–5 micron sections were stained with hematoxylin and eosin (H/E) for histological assessment by a board-certified veterinary pathologist (JMS). The pathologist was blinded to treatment until her assessments had been completed. For further analysis of the female reproductive tract, Congo red staining and Masson’s trichrome staining were performed on 10 OH_Est treated mice and 10 control mice, all from the UM cohort.

Lesions recorded as present (1) or absent (0) included the following: all neoplasms; respiratory epithelial hyperplasia; atrial thrombosis; fibro-osseous lesion of bone; splenic angiectasis; seminal vesicle adenitis; encapsulated or mineralized fat in the abdomen; “cold follicles” of the thyroid (defined as dilated colloid-filled follicles with flattened epithelium), and thyroid follicular hyperplasia. Lesions scored on a scale of 0 (absent), 1 (minimal to mild) and 2 (moderate to severe) included bladder dilation and islet cell hyperplasia. Other lesions were graded on a 0 (absent) to 4 (severe) scale, with a score of 1 indicating minimal; 2 indicating mild; and 3 and 4 indicating moderate and severe lesions, respectively. These lesions included eosinophilic crystalline pneumonia; cardiomyopathy; glomerulonephropathy; macrovesicular cytoplasmic vacuolation of the hepatocytes (lipidosis, presumptive); microvesicular cytoplasmic vacuolation of the hepatocytes (lipidosis, presumptive); hepatic necrosis/hepatocellular degeneration; hepatocellular inflammatory cell infiltration; pancreatic exocrine atrophy; pancreatic lymphoid accumulations; adrenal pigment, atrophy, and degeneration (coded as adrenal degeneration); ovarian atrophy, lipofuscinosis, and degeneration (coded as ovarian degeneration); testicular atrophy/degeneration; and uterine cystic endometrial hyperplasia. For paired organs, both organs were scored (when present) and the average score was recorded. Autolysis in some organs (most commonly adrenal glands, kidney, pancreas, and spleen) from some mice precluded scoring, and scoring also was not performed if the organ was not present or only a small section of the organ was obtained (most commonly adrenal gland and thyroids). Scoring was performed as previously described [2] including criteria previously developed and validated to assess lesions of the kidney, heart, liver, and lung in aged mice [7].

For females only, the reproductive tract was further evaluated for uterine or ovarian thrombosis (present/absent); ovarian cyst (present/absent); hyaline material expanding the myometrium of the uterus (present/absent); and angiectasis (scored 0–4).

Vascular lesions and adrenal neoplasms were reviewed with a second board-certified veterinary pathologist (DMI) for agreement and consistency of diagnosis. As in a previous study [5], adrenal cortical neoplasms in this study were diagnosed on the basis of the degree of compression of the adjacent adrenal parenchyma, disruption of the normal cortical architecture, and/or extension above the normal contour of the adrenal surface.

### Statistics

Control mice were compared separately to Cana_16 mice and to OH_Est mice. For each lesion, statistical significance was based on a two-factor ANOVA, with sex, treatment, and interaction factors evaluated. When the *p*-value for Interaction did not meet the criterion *P* < 0.05, the *P*-value for the treatment term was used to infer drug effect on the incidence or severity of the lesion. When the p-value for Interaction was *P* < 0.05, this was taken as evidence for a drug effect that was different depending on the sex of the mice. No adjustments were made for possible multiple-comparison effects.

## Results

### Cause of death is not significantly affected in male or female mice by either 16α-hydroxyestradiol started at 12 months of age or Canagliflozin started at 16 months of age

Table 1 shows the cause of death for male and female mice in this study. Neoplasia was the most common cause of death for mice of both sexes in all groups, and a neoplastic process accounted for the cause of death in 72% of mice, including 77% of female mice and 65% of male mice (85% of female mice and 72% of male mice for which a cause of death was identified). The most common causes of death were hematopoietic neoplasia (particularly in females), lung carcinoma (particularly in males), mammary carcinoma (in females), hepatocellular carcinoma (in males), hemangiosarcoma (in females), and pituitary neoplasia (in females). Several mice had more than one neoplasm contributing to their cause of death. There were no significant differences in cause of death for either sex following treatment with OH_Est or 16_Cana.

**Table 1:** Cause of death in control mice, mice treated with OH_Est, and mice treated with Cana_16

Cause of death	Female controls (*n* = 30)	Male controls (*n* = 15)	Female OH_EST (*n* = 29)	Male OH_EST (*n* = 29)	Female 16_Cana (*n* = 30)	Male 16_Cana (*n* = 31)	Total
NEOPLASTIC							
Hemangiosarcoma	0	0	1	1	3	0	5
Hepatocellular carcinoma	1	1	0	1	0	2	5
Hematopoietic neoplasia	10	1	13	6	6	7	43
Lung carcinoma	2	1	1	2	2	7	15
Mammary carcinoma	1	0	3	0	5	0	9
Pituitary neoplasm	1	1	4	0	0	0	6
Squamous cell carcinoma	0	0	0	2	1	0	3
Other neoplasia	2	4	1	6	4	1	18
Multiple neoplasms	4	0	1	1	3	5	14
Total	21	8	24	19	24	22	118
NONNEOPLASTIC							
Uterine thrombosis	1	n/a	0	n/a	2	n/a	3
Amyloidosis	0	0	0	1	1	0	2
Atrial thrombosis	1	2	0	0	0	1	4
Cardiomyopathy	0	0	0	0	0	1	1
Glomerulopathy	0	0	0	1	0	0	1
Inflammation	1	0	2	0	0	0	3
Bladder obstruction	0	2	0	0	0	3	5
Liver necrosis	0	0	0	3	0	0	3
Multiple conditions	2	1	1	1	1	3	9
Total	5	5	3	6	4	8	31
UNKNOWN	4	2	2	4	2	1	15

### Effects of OH_Est and Cana_16 on lesion frequency and severity

In addition to recording lesions considered most likely to represent the cause of death, we also recorded the presence or absence of a variety of neoplastic, degenerative, and other lesions noted on gross and histologic examination, even if these were not considered to be a likely cause of death. Although there were no statistically significant changes induced by either drug in inferred cause of death, each drug did lead to multiple changes in the frequency or severity of some of these lesions detectable at necropsy. Table 2 presents all lesions for which the drug effect was either significant (*P* < 0.05) or suggestive (0.05 < *P* < 0.1, as shown) for one or both interventions. In each case, the comparison was to untreated control mice from the same, C2020, cohort. Categorical lesions are shown first, followed by lesions graded on a severity scale of 0–4. Hemangiosarcoma, for example (top line) was noted in 0/15 control males and 0/30 control females, but in 2/31 Cana_16 males, 5/30 Cana_16 females, 4/29 OH_Est males, and 4/29 OH_Est females. Two-factor ANOVA was used to compare each drug-treated group to controls, yielding p-values for drug effect, sex effect, and [Drug x Sex] interaction. The table shows *p*-values for drug effect when the interaction term is not significant (at *P* < 0.05) and for the interaction term when *P* < 0.05. In this context, a significant interaction term suggests that the extent, or direction, of the drug effect depends on the mouse’s sex. For graded lesions, the table shows mean levels and standard error of the mean, for each pertinent group. The prevalence of similar lesions in control mice from previous ITP studies is presented in Supplemental Table 1.

**Table 2:** Categorical and graded lesions present on histological examination, even if not considered a likely cause of death

Categorical lesion	Control (M, F)	Cana (M, F)	OH-Est (M/F)	Control vs Cana	Control vs OH-Est	Direction of change
Hemangiosarcoma	0/15, 0/30	2/31, 5/30	4/29, 4/29	*P* = 0.02	*P* = 0.01	Both drugs increased prevalence
Pituitary neoplasia	1/15, 2/30		1/28, 8/27		Int *P* = 0.05	Increased in females
Lung adenocarcinoma	4/14, 4/30		1/29, 2/28		*P* = 0.02	Decreased prevalence
Liver adenoma	4/14, 1/28	1/30, 2/29	2/29, 0/27	Int *P* = 0.02	*P* = 0.02	Both drugs decrease prevalence
Adrenal cortical neoplasia	5/13, 0/25	6/29, 2/29	3/27, 2/26	(Int *P* = 0.08)	Int *P* = 0.008	Down in males, up in females
Hepatocellular carcinoma	1/14, 4/29	9/30, 0/29		Int *P* = 0.01		Up in males, down in females
Thyroid hyperplasia	0/12, 4/23		4/18, 0/19		Int *P* = 0.01	Up in males, down in females
Atrial thrombus	3/15, 1/30		0/29, 0/29		Int *P* = 0.03	Down in males
Encapsulated mass	1/15, 7/30		1/29, 1/27		(*P* = 0.06)	Down in females
Fibro-osseous bone lesion	0/14, 2/28		16/27, 14/23		*P* < 0.0001	Increased prevalence
Hyaline material uterus	NA, 4/30	NA, 5/30	NA, 24/30			
Hematopoietic neoplasia (HPN)						
Cranial HPN	0/15, 4/30		2/28, 0/27		Int *P* = 0.04	Down in females
Uterine HPN	NA, 11/29	NA, 4/29	NA, 4/27	*P* = 0.04	(*P* = 0.052)	Both drugs decrease prevalence
Liver HPN	2/13, 11/27	9/30, 5/30		Int *P* = 0.04		Up in males, down in females
Graded lesion	Control	Cana	OH-Est	Control vs Cana	Control vs OH-Est	Direction of change
Cardiomyopathy	1.9 ± 0.15		1.6 ± 0.12		(*P* = 0.08)	Less severe
Glomerulonephritis	1.9 ± 0.14		1.7 ± 0.11		*P* = 0.03	Less severe
Pancreas: exocrine atrophy	1.1 ± 0.17		0.7 ± 0.73		(*P* = 0.051)	Less severe
Uterus: cystic endometrial hyperplasia (Females)	1.8 ± 0.16		2.9 ± 0.16		*P* = 0.0001	More severe
Adrenal degeneration	2.2 ± 0.13		2.6 ± 0.10		*P* = 0.007	More severe
Ovarian atrophy (female)	3.6 ± 0.16	2.9 ± 0.17		*P* = 0.008		Less severe

*NA* not applicable, i.e., uterus not present in male mice

Cana_16 lowered the risk of uterine HPN and lowered the severity of ovarian atrophy, lesions limited to females. In contrast, Cana_16 was associated with an increased prevalence of hemangiosarcoma in both sexes. The other four Cana_16 effects were all sex-specific (significant interaction term). Liver HPN and hepatocellular carcinoma were increased in males but decreased in females given Cana_16. Adrenal cortical neoplasia and liver adenomas decreased in males but increased in females. This complex set of changes suggests a high degree of sexual dimorphism in Cana_16 effects on late-life pathology.

OH_Est led to significant, sex-independent, changes in 4 lesions, sex-specific changes in frequency of 5 others, and changes in severity of 5 lesions. Four of these 14 tabulated changes were merely suggestive, with 0.05 < *P* < 0.1, and are included here to minimize Type II errors. In some cases, the agent lowered risk in one or both sexes: intracranial HPN, lung adenocarcinoma, atrial thrombus, liver adenoma, uterine HPN, and encapsulated adipose abdominal mass. In some cases, the direction of effect was sex-specific: adrenal neoplasia was diminished in males but increased in females, while the prevalence of thyroid hyperplasia showed the opposite pattern. OH_Est was associated with an increased frequency of hemangiosarcoma and fibro-osseous bone lesions (both sexes) and pituitary neoplasia (females only). OH-Est increased the severity of uterine cystic endometrial hyperplasia and adrenal degeneration, and led to lower severity of cardiomyopathy (suggestive at *P* = 0.08), glomerulonephritis, and pancreatic endocrine atrophy (at *P* = 0.051). Thus, OH_Est, like Cana_16, leads to a complex set of organ-specific and, in some cases, sex-specific effects on late-life pathology.

**Fig. 1:**
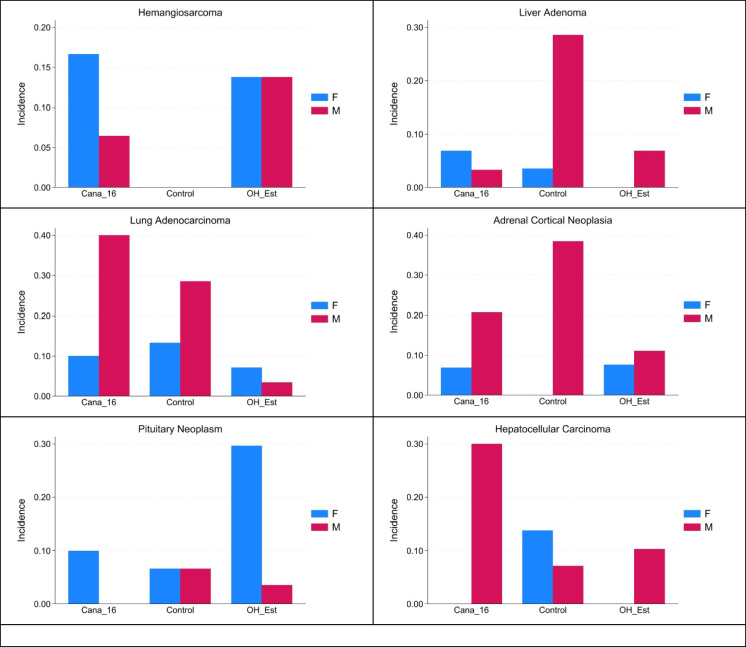
Prevalence of selected proliferative lesions in mice treated with OH_Est or with Cana_16 compared to control groups, by sex. This represents a graphical depiction of the data in Table 2, and shows the prevalence of **A** hemangiosarcoma; **B** liver adenoma; **C** lung adenocarcinoma; **D** adrenal cortical neoplasia; **E** pituitary neoplasia; and **F** hepatocellular carcinoma

Figure 1 illustrates a sampling of these observations graphically. Hemangiosarcoma (upper left) is not seen in controls, but its prevalence is increased significantly in both drug treatment groups in both sexes. Liver adenoma (upper right) is rarely present in females but is reduced by both drugs in males. Lung adenocarcinoma (middle left) is less common in both male and female OH_Est treated mice; whereas, the prevalence of adrenal cortical neoplasia decreases in males but increases in females OH_Est treated mice. Pituitary neoplasia (lower left) is rarely seen in male and female controls, but its frequency is increased in female OH_Est treated mice. The prevalence of hepatocellular carcinoma (lower right) shows the opposite pattern in OH_Est mice, increasing in males and decreasing in females. Significance tests for each of these lesions are included in Table 2.

**Fig. 2:**
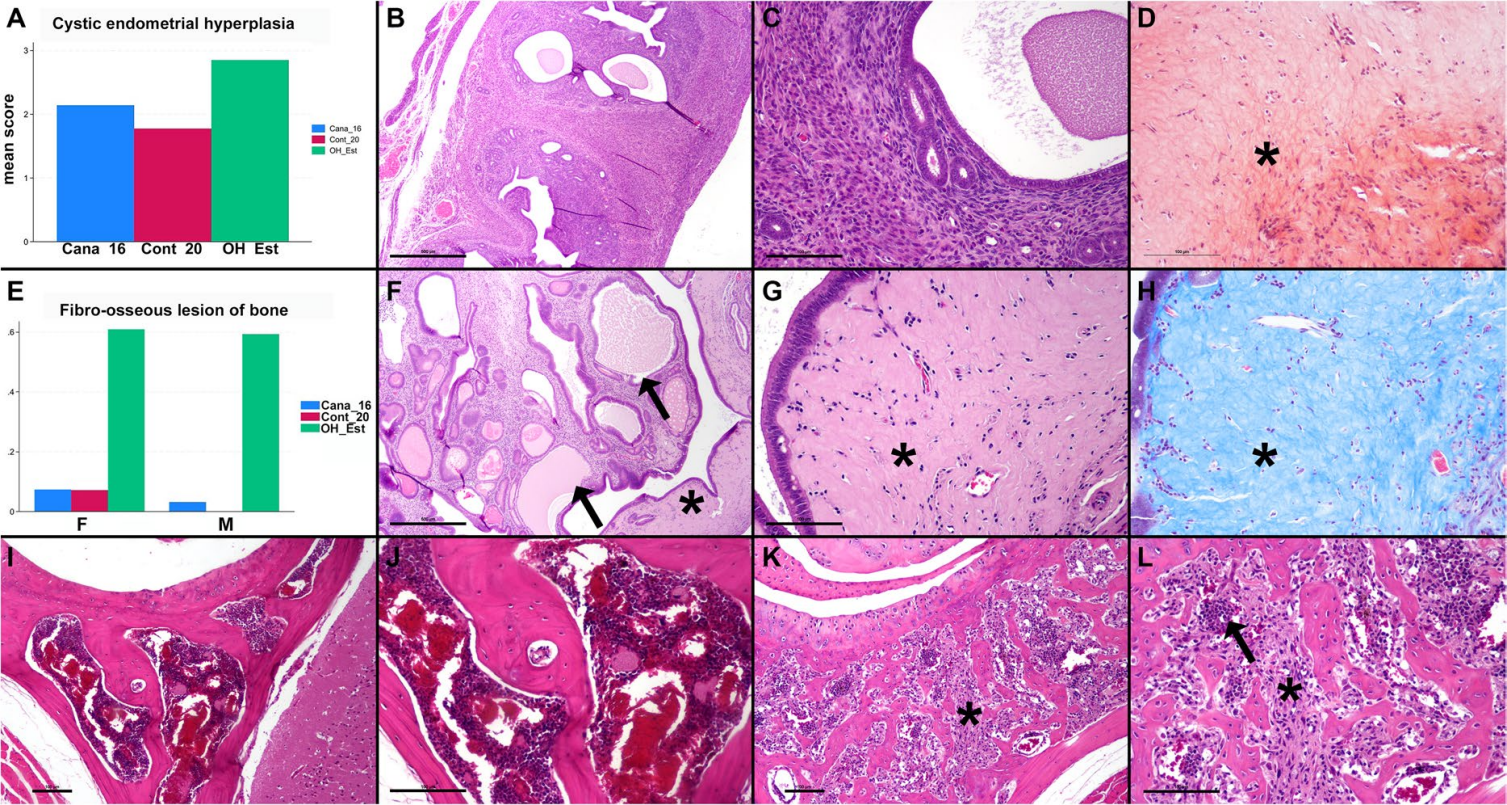
OH_Est associated lesions in mice. **A** The severity of cystic endometrial hyperplasia was dramatically increased in OH_Est treated mice (*P* = 0.0001). **B** Uterus from a control mouse, showing mild proliferation of the endometrial glands and mildly increased dilated glands (cystic endometrial hyperplasia). Hematoxylin and eosin (HE), bar = 500 microns. **C** Higher magnification of the uterus in (**B**), showing the normal endometrial and myometrial tissue surrounding the glands. HE, bar = 100 microns. **D** Congo red staining of the glassy, eosinophilic tissue surrounding the endometrial glands in OH_Est treated mice. **E** The prevalence of fibro-osseous lesions (y axis) was also increased in OH_Est treated male and female mice (*P* < 0.0001). **F** Increased cystic endometrial hyperplasia in an OH_Est treated mouse, showing dilated glands (arrows) with densely staining columnar cells or attenuated epithelium. There is eosinophilic, acellular glassy material expanding the stroma surrounding the glands (asterisk). HE, bar = 500 microns. **G** Higher magnification of the eosinophilic, acellular glassy material expanding the stroma surrounding the glands (asterisk). HE, bar = 100 microns. **H** Masson’s trichrome staining of the glassy, eosinophilic tissue surrounding the endometrial glands in OH_Est treated mice. **I** Normal bone and bone marrow of the bone associated with the temporomandibular joint (TMJ) in a control mouse. HE, bar = 100 microns. **J** Higher magnification of the bone in I showing the normal appearance of the bone marrow. HE, bar = 100 microns. **K** Fibro-osseous lesion in an OH_Est treated mouse at the level of the TMJ, showing replacement of the marrow with fibrovascular stroma (asterisk). **L** Higher magnification of the bone in (**K**). Normal hematopoietic cells are indicated with an arrow. HE, bar = 100 microns

Female mice treated with OH_Est also had an increased tendency to accumulate glassy, eosinophilic, acellular material in the stroma of the endometrium and myometrium of the uterus compared to mice treated with Cana_16 and control mice. This material stained blue on Masson’s trichrome staining and orange-red on Congo red staining (but is not birefringent under polarized light) and was noted in 24/30 OH_Est treated mice, 5/30 Cana_16 treated mice, and 4/30 control mice (Fig. 2). Figure 2 shows additional examples of lesions that were significantly increased in OH_Est treated mice, including fibro-osseous lesions of bone in male and female mice, increased hyaline material expanding the uterine stroma, and cystic endometrial hyperplasia in female mice.

All lesions are shown for which *P* < 0.1 for one or both drugs, when compared to control mice. *P*-values are shown in parentheses when 0.1 < *P* < 0.05. Analyses used two-factor ANOVA, which drug and sex as factors, with the interaction term included. Each drug was evaluated separately against control. *P*-values stated as “Int P” were for the interaction term and indicate drug effects that differed between male and female mice for categorical lesions. Graded lesions were recorded on a 0–4 scale. There were no sex-specific effects (i.e., no significant Interaction terms) for the graded lesions. For categorical lesions, entries for each sex show [lesions present]/[mice examined]. For graded lesions, entries show mean ± standard error of the mean.

## Discussion

This end-of-life necropsy study was stimulated by the recent ITP report [1] of lifespan effects of OH_Est and Cana_16 in UM-HET3 mice. Both drugs were found to extend male lifespan but to lead to a significant decline in the lifespan of female mice. Our pathology study thus paid special attention to the possibility of sex-specific effects on late-life lesions.

In the original report, Cana given from 16 months of age produced a 14% increase (*P* = 0.004) in lifespan of male mice but a 6% decrease in lifespan of females (*P* = 0.03). At the same dose, Cana administered from 7 months of age [2] had led to a 14% increase in median lifespan of males and to a significant increase in the proportion of mice alive at the 90th percentile of the joint survival distribution, a surrogate measure for “maximal” lifespan. Cana administered from 7 months of age did not, however, produce a significant change in lifespan of female mice. A necropsy study of male and female mice, using tissues taken at 22 months of age from mice given Cana from 7 months [5], found diminished incidence of multiple lesions in Cana-treated male mice, of which only one, pancreatic exocrine atrophy, was also reduced in females. Thus, Cana seems to be potentially toxic to females when administered to older mice (e.g., from 16 months), but neutral if given from younger ages. This discrepancy may well reflect age-specific, sex-specific differences in drug conjugation and/or elimination, in that blood concentrations in older female mice are approximately 20-times higher than in young male mice [1].

Tabulation of cause of death data can, in principle, develop evidence that changes in median longevity reflect higher or lower incidence of some specific lethal disease, for example, a hypothetical increase in lifespan due to reduction in incidence of lethal hematopoietic malignancy. Our cause of death data (Table 1) showed no evidence for a significant effect of either Cana_16 or OH_Est in the percentage of deaths attributable to any one form of lethal illness. Indeed, no such anomaly has been seen for any of the end-of-life necropsy studies in the ITP’s history [2, 4, 6]. This is consistent with the idea that anti-aging drugs may lead to equivalent postponement of multiple, perhaps all, forms of lethal illness in parallel, but may also reflect the relatively low statistical power of our current and previous necropsy series. A lesion that leads to death of, for example, 10% of controls would only be noted in 6 mice of a series of 30 males plus 30 females, and even an 83% reduction (from 6 deaths to 1 death, *P* = 0.052) would not achieve statistical significance. Of the lethal illnesses listed in Table 1, only HPN was seen in more than 10% of the control mice.

Our tabulation of “incidental” lesions, i.e., lesions that were detectable but did not necessarily contribute to the death of the mouse, revealed some evidence for Cana_16 effects (Table 2). Of the seven lesions for which Cana_16 had a significant effect, one showed increased frequency in both sexes, 3 showed a decline in prevalence or severity that was not sex-specific, and 3 others showed a sex-specific effect. Of the three with sex-specific effect, only adrenal cortical neoplasia was increased in female mice; it was detected in 0/25 control females and 2/29 Cana_16 females. The study of incidental pathology thus revealed effects of Cana_16 on multiple tissues but does not provide much insight into the basis for the relatively short lifespan of treated females. It seems plausible that the earlier death of Cana_16 females may reflect subtle metabolic or physiologic abnormalities not detected by standard histopathology. The ITP is currently evaluating lifespan effects of Cana given at lower doses, or for briefer intervals (from 7 to 20 months) to female mice, in the hopes of diminishing late-life toxicity and potentially revealing latent benefits, in females, that could be masked by late-life toxic effects.

The lifespan study of 16α-hydroxyestradiol (OH_Est) was motivated by the observation of male-specific metabolites of 17α-estradiol. It was hypothesized that these male-specific metabolites [8] might account for the male-specific lifespan benefit. OH_Est did indeed lead to significant lifespan extension in males, but, contrary to hypothesis, led to shorter lifespan in female UM-HET3 mice [1]. We found no evidence in either sex for alteration of the proportion of lethal illnesses (Table 1), a finding limited by the same lack of statistical power discussed above. There were, however, 12 varieties of incidental lesion that were modified (at *P* < 0.05) in incidence or severity by OH_Est, and 4 others for which *P* < 0.1, listed in Table 2. Of these 16, five showed sex-specific differences in lesion prevalence, and only two of these, pituitary neoplasia and adrenal cortical neoplasia, were elevated specifically in female mice.

Pituitary neoplasia was noted in 2/30 (7%) of control female mice but in 8/27 (30%) of OH_Est females. Pituitary neoplasia was noted in 1/15 control males and 1/28 OH_Est males (7% vs 4%). It thus appears that females are particularly susceptible to OH_Est effects on pituitary neoplastic disease. Further studies, potentially including hormonal assays or immunohistochemistry, would be needed to learn more about the specific types of pituitary cells transformed in these mice, and to evaluate whether hormones secreted by pituitary tumors could have contributed to ill health in the OH_Est treated females. Pituitary and other neoplasms including reproductive and bone tumors have been associated with long-term administration of 17-β-estradiol and its esters in mice and rats [9–13]. In previous studies, mice treated with 17-β-estradiol also developed cystic endometrial hyperplasia and acellular hyaline material expanding the uterine stroma, which were also increased in our OH_Est treated mice [11, 14, 15].

Fibro-osseous bone lesions were dramatically increased in OH_Est mice of both sexes and were seen in 2/42 (5%) of control mice but in 30/50 (60%) of treated mice. Fibro-osseous lesion is a relatively common age- and strain-related finding in female mice and is characterized by replacement of the bone marrow, cancellous, and cortical bone by fibrovascular stroma, particularly in the skull, vertebrae, and long bones of the femur and tibia [16]. This lesion can be associated with cystic endometrial hyperplasia, ovarian cysts, ovarian atrophy, hydrometra, and degenerative joint disease in female mice. It does not appear to be associated with renal or parathyroid dysfunction or with myelofibrosis and is an uncommon spontaneous lesion of male mice [16, 17]. 17-β-estradiol has been associated with fibro-osseous lesion of bone in male and female mice but was not associated with an increased risk of neoplasms [18]. In future studies, it would be interesting to further characterize this lesion by examining additional bone locations, evaluating for concurrent changes such as degenerative joint disease, and potentially testing for evidence of increased frailty in mice treated with OH_Est.

Scores for a spectrum of lesions of the adrenal glands including increased pigment deposition, cortical atrophy, and cystic degeneration were also higher in OH_Est mice than in controls. Increased accumulation of brown pigment consistent with ceroid in the adrenals has been previously reported in mice treated with ethinyl estradiol [11, 19, 20] and may contribute to the higher scores in this study.

In the present study, both OH_Est and Cana were associated with a significant increase in the prevalence of hemangiosarcoma. This may be related to the absence of this lesion in control mice, which is unusual in an ITP study. In recent longitudinal studies performed by the ITP, hemangiosarcoma has been identified in 6/60 and 6/58 control mice [2, 21], which is similar to the frequency observed in Cana-16 and OH_Est treated mice in the current study. For this reason, the apparent increase in hemangiosarcoma seen in the current study should be interpreted with caution. Also, in the present study, some differences were noted in the frequency of hematopoietic neoplasia in particular organs (female reproductive, liver, intracranial); however, the overall prevalence of hematopoietic neoplasia was not significantly different between treatment groups, so the implication of this finding is unclear.

A previous cross-sectional study identified several histologic parameters that were improved in male mice treated with Canagliflozin from 7 to 22 months of age, including age-related cardiomyopathy, glomerulonephropathy, and pancreatic exocrine atrophy [5]. It is difficult to compare the results of this longitudinal study (in which male mice treated with Cana were older than controls at the time of necropsy given the increased lifespan) with the previous cross-sectional study. One finding that was similar in both studies was a decreased frequency of adrenocortical neoplasms in male mice treated with Cana. In the previous cross-sectional study, males treated with Cana had significant bladder dilation, which was not noted in the current study. Cross-sectional studies of Cana_16 and OH_Est may help elucidate some of the findings presented here and allow for comparison of tumor incidence and scores of age-related degenerative lesions at the same time point.

In summary, both Cana_16 and OH_Est modify incidence and/or severity of age-dependent lesions in multiple tissues, including bone, pituitary, brain, lung, adrenal gland, liver, and uterus, including a mixture of protective and harmful effects. Although some of the drug effects show sex-specific dimorphism, none of them provide a compelling explanation for the harmful effects of these drugs on female lifespan nor their ability to increase lifespan in male mice. Increases in pituitary neoplasms in OH_Est treated female mice identified in this study deserve further attention as a potential cause of ill health in this model.

## Supplementary Information

Below is the link to the electronic supplementary material.

Supplementary file1 (DOCX 15 KB)

## Data Availability

Data is available upon request.
