# Supplementary material for: End-of-life pathology in UM-HET3 mice treated with 16 α‑hydroxyestradiol or late‑start canagliflozin
Source: GeroScience. 2025 Jul 2;48(2):1787–97. doi: 10.1007/s11357-025-01741-3 (PMC12972294; doi:10.1007/s11357-025-01741-3)
Supplement: Supplementary file 1 — Supplementary file1 (DOCX 15 KB) [file 11357_2025_1741_MOESM1_ESM.docx]

Table 1. Histologic lesions present in control mice from this study versus control mice from two previous ITP studies (Canagliflozin and Glycine longitudinal).

g = Glycine study (reference 22)

c = Canagliflozin study (reference 2)

| **Organ** | **Lesion** | **Control Cases**  **N=45** | **Control Cases^g^**  **N=59** | **Control cases^c^**  **N=60** |
| --- | --- | --- | --- | --- |
|  | **Categorical lesions** |  |  |  |
| Adipose | Mineralization of mesenteric adipose tissue | 8 | 7 | 7 |
| Adrenal | Neoplasm | 5 | 7 | 3 |
| Atrium | Thrombus | 4 | 3 | 5 |
| Liver | Adenocarcinoma | 5 | 5 | 4 |
| Lung | Adenocarcinoma | 8 | 13 | 15 |
| Mammary | Neoplasm | 5 | 1 | 6 |
| Pituitary | Neoplasm | 3 (1M, 2F) | 2 (1M, 1F) | 3 (3F) |
| Various | Hemangiosarcoma | 0 | 6 | 6 |
| Various | Hematopoietic neoplasia | 18 | Not specified* | 20 |
|  | **Graded lesions** |  |  |  |
| Heart | Cardiomyopathy | 1.9 ± 0.15 | 1.5 | 1.6 |
| Kidney | Glomerulonephropathy | 1.9 ± 0.14 | 1.7 | 1.8 |
| Pancreas | Pancreas: exocrine atrophy | 1.1 ± 0.17 | 0.5 | 0.8 |
| Uterus | Uterus: cystic endometrial hyperplasia (Females) | 1.8 ± 0.16 | 1.9 | 2.1 |
| Adrenal gland | Adrenal pigment, atrophy and degeneration | 2.2 ± 0.13 | 1.6* | 1.9 |
| Ovary | Atrophy, lipofuscinosis, and degeneration | 3.6 ± 0.16 | 3.4 | 3.4 |

*For the glycine longitudinal study, hematopoietic neoplasms were reported by organ rather than as a total number of animals affected, and the grading criteria used to score age-related changes of the adrenal gland was slightly different than that used for the other two studies.
